# Openness to experience predicts dopamine effects on divergent thinking

**DOI:** 10.1017/pen.2019.3

**Published:** 2019-07-26

**Authors:** Wiebke Käckenmester, Antonia Bott, Jan Wacker

**Affiliations:** Institute for Psychology, Universität Hamburg, Hamburg, DE Germany

**Keywords:** openness to experience, dopamine, divergent thinking

## Abstract

Individual differences in trait levels of openness to experience and creativity have been theoretically linked to dopamine function. However, empirical evidence for this assumption is scarce, especially for causal connections. The present study aims to directly assess the influence of dopamine activity on the established association between openness to experience and divergent thinking (i.e., an index of creativity). We hypothesized that manipulating dopamine activity alters the relationship between self-reported openness to experience and ideational fluency and flexibility. In a placebo-controlled between-subjects design, 193 healthy male volunteers completed four divergent thinking tasks after they received either the dopamine-receptor blocker sulpiride (200 mg) or a placebo. The data revealed an interaction such that openness to experience was more positively associated with divergent thinking in the dopamine blocker group (*r* = 0.304) than in the placebo group (*r* = −0.002). Specifically, highly open individuals in the dopamine blocker group reached the highest divergent thinking scores. Thus, sulpiride administration selectively affected divergent thinking as a function of trait levels of openness to experience. Although somewhat limited by the unexpected absence of the association between openness to experience and divergent thinking in the placebo group, the present study provides novel evidence for an association between dopamine activity and both openness to experience and divergent thinking.

Openness to experiences has been prominently described as “the breadth, depth, and permeability of consciousness” (McCrae & Costa, 1997, p. 826). Open people notice and appreciate novel, complex, and unusual information in a variety of everyday experiences (DeYoung, Quilty, Peterson, & Gray, 2014; McCrae, 1994). Conceptually and empirically, individual differences in openness to experience have been closely related to creativity. Some have even proposed creativity as an alternative label for the fifth factor of personality (Johnson, 1994; Saucier, 1992); others viewed creativity as a central characteristic of openness to experience, including the ability to make remote and unusual associations (Costa & McCrae, 1992). Still others regard openness to experience as a psychological factor that promotes the acquisition of cognitive creative potential and facilitates everyday creative activities (Jauk, 2019). Empirically, openness to experience has been positively associated with self-reported creative activities (Batey, Chamorro-Premuzic, & Furnham, 2010; Jauk, Benedek, & Neubauer, 2014; Wolfradt & Pretz, 2001), creative achievements (Feist, 1998; Kaufman et al., 2015; King, Walker, & Broyles, 1996), and performance in creative thinking tasks, such as remote consequences and divergent thinking (e.g., Jauk et al., 2014; McCrae, 1987). The association between openness to experience and divergent thinking has been frequently studied and empirically well established (Puryear, Kettler, & Rinn, 2017). Requiring the ability to generate numerous, various, and original ideas for a given scenario, usually either in the verbal domain (e.g., list various uses for a brick; Guilford, 1967) or in the figural domain (e.g., draw objects that complete given lines; Torrance, 1972), divergent thinking has been viewed as one of the most essential cognitive prerequisites of creativity (Guilford, 1957).

## 1. The dopaminergic basis of openness to experience and creativity

Recent years have seen an increase in work on the neurobiological basis of openness to experience and creativity (for a brief recent review, see Jauk, 2019), at least partly inspired by a series of reviews and theoretical articles by Colin DeYoung and colleagues (e.g., DeYoung, 2013, 2014; DeYoung, Peterson & Higgins, 2005): DeYoung et al. (2005) initially suggested that openness to experience is based on individual differences in cognitive exploration, which in turn partly results from individual differences in dopaminergic neurotransmission. Similar assumptions have been made for the closely related creativity dimension (DeYoung, 2013).

The following observations provide initial indirect support for these ideas: First, openness to experience (Peterson & Carson, 2002; Peterson, Smith, & Carson, 2002) and creative achievement (Carson, Higgins, & Peterson, 2003) have been reported to negatively correlate with latent inhibition, a low-level cognitive phenomenon relevant for shielding formerly ignored information from further processing (i.e., arguably an indicator of “permeability of consciousness”) and sensitive to dopaminergic drugs (Swerdlow et al. 2003; Weiner & Feldon, 1987; Weiner, Shadach, Tarrasch, Kidron, & Feldon, 1996). Second, openness to experience has been shown to correlate with increased functional connectivity within dopamine-rich mesocortical networks (Passamonti et al., 2015). Third, divergent thinking has been associated with decreased dopamine D2 receptor density in a very small sample of *n* = 14 (de Manzano, Cervenka, Karabanov, Farde, & Ullén, 2010) and with increased mean diffusivity in dopamine-rich brain regions (Takeuchi et al., 2015). Fourth, creative thinking has been associated with eye blink rate (i.e., an indicator of dopamine activity; Akbari Chermahini & Hommel, 2010). Finally, it has been demonstrated that Parkinson’s disease patients increasingly engaged in creative activities with the introduction of dopaminergic medication and reduced these activities after dose reductions (Lhommée et al., 2014). When treated with dopamine-increasing medication, Parkinson’s disease patients have further demonstrated enhanced divergent thinking performance compared with non-medicated healthy controls (Faust-Socher, Kenett, Cohen, Hassin-Baer, & Inzelberg, 2014).

Although these converging findings are encouraging, more direct evidence from pharmacological studies targeting dopamine activity in larger samples is needed. To our knowledge, only one published study has addressed connections between dopamine, creativity, and personality traits using a pharmacological approach in healthy participants: In a small sample (*n* = 33), Gvirts and colleagues (2017) reported diminished verbal divergent thinking under the dopamine reuptake inhibitor methylphenidate (20 mg) versus placebo only in participants high in novelty seeking (i.e., a trait moderately associated with both openness to experience and divergent thinking; Goclowska, Ritter, Elliot, & Baas, 2018). Whether analogous modulating effects of dopamine can be demonstrated for openness to experience remains to be tested.

## 2. The present study

The present study provides an initial direct test of the hypothesis that openness to experience modulates the effects of a pharmacological manipulation of dopamine on measures of divergent thinking. Groups of healthy males received either the dopamine receptor blocker sulpiride or a placebo prior to performing four divergent thinking tasks. Openness to experience was measured beforehand and independently of the pharmacological manipulation. The main prediction of an interaction between openness to experience and substance group (sulpiride vs. placebo) was based on the general observation that traits thought to be associated with individual differences in dopamine typically modulate the effects of pharmacological manipulations of dopamine on variables associated with the trait in question. Although this pattern remains to be demonstrated for openness to experience, it has already been documented quite consistently for extraversion, that is, a trait likewise hypothesized to be associated with brain dopamine (Depue & Collins, 1999; Wacker & Smillie, 2015). While openness to experience is thought to be based on individual differences in cognitive exploration resulting from dopaminergic variability in a mesocortical pathway underlying salience processing, extraversion is thought to be based on individual differences in behavioral exploration resulting from dopaminergic variability in a mesolimbic pathway underlying reward processing (DeYoung et al., 2005). Supporting the later suggestion, extraversion has been connected to individual differences in electroencephalogram (EEG) and functional magnetic resonance imaging indicators of reward processing (Müller et al. 2014; Wu, Samanez-Larkin, Katovich, Knutson, 2014) and responsivity to dopaminergic drugs (Depue, Luciana, Arbisi, Collins, Leon, 1994; Depue, 1995). Furthermore, pharmacological manipulations have been shown to alter the association between extraversion and EEG correlates of reward processing (Mueller et al. 2014; Wacker, Mueller, Pizzagalli, Hennig, Stemmler, 2013). Because openness to experience and extraversion are typically moderately correlated and systemic pharmacological manipulations cannot specifically target either the mesocortical or the mesolimbic dopamine system, potential effects of extraversion were statistically controlled in the current study. The same presumption holds for intelligence, which has been regularly found to be moderately correlated with both divergent thinking and openness to experience (Ashton, Lee, Vernon, Jang, 2000; Austin, Deary, & Gibson, 1997; Benedek, Jauk, Sommer, Arendasy, & Neubauer, 2014; Harris, 2004; McCrae, 1993; Nussbaum & Silvia, 2011).

## 3. Methods

### 3.1 Participants

A total of 210 healthy male volunteers participated in the present experiment. The study was part of a larger research project investigating the effects of dopamine on behavioral measures. Participants were recruited on social media platforms, job fair websites, and on campus. They provided written informed consent and received monetary compensation (€70) or course credit for their 6-h involvement in the research project. The study was approved by the Ethics Committee of the German Society for Psychology. Accordingly, the authors assert that all procedures contributing to this work comply with the ethical standards of the relevant national and institutional committees on human experimentation and with the Helsinki Declaration of 1975, as revised in 2008. Inclusion criteria were male gender, right-handedness, physical and mental health, and age between 18 and 35 years. The sample was restricted to male participants to control for sex-specific differences that might interfere with substance effects (e.g., due to the female hormonal cycle). In a pretesting session, the absence of psychiatric disorders was verified using a standardized clinical interview (Mini-DIPS; Margraf, 1994). Indications of psychiatric disorders as well as hypertension (blood pressure higher than 140/90) led to exclusion. Further self-reported exclusion criteria were the intake of any prescription medication or illegal drugs during the last 3 months, and habitual smoking of more than 10 cigarettes per week. In total, 17 participants were excluded from this study because they reported a first language other than German (*n* = 10), refused to comply with task instructions (*n* = 2), were not able to swallow the capsule (*n* = 2), arrived too late for medication intake (*n* = 1), had already eaten on the study day (*n* = 1), and did not complete the personality questionnaire due to technical failure (*n* = 1). The final sample reported here consisted of 193 males (mean age = 25.8, *SD* = 3.9; *n* = 95 in the sulpiride group and *n* = 98 in the placebo group). Of them, 76% were university students (7.7% psychology students). As intended, statistical power was therefore >0.80 to detect correlations of *ρ* = 0.30 (*α* = 0.05) within each of the two substance groups.

### 3.2 Materials and tasks

#### 3.2.1 Pharmacological manipulation

In a placebo-controlled, double-blind between-subjects design, participants orally ingested either 200 mg sulpiride or a placebo. Both substances were delivered in capsules matched for weight and color to assure double-blindness to participants’ experimental conditions. Sulpiride is classified as substituted benzamide that predominantly acts as selective D2-receptor antagonist (Mauri, Bravin, Bitetto, Rudelli, & Invernizzi, 1996). While showing high affinity to both pre- and postsynaptic D2 receptors (Missale, Nash, Robinson, Jaber, & Caron, 1998), the substance appears to lack significant effects on other receptor types (e.g., histaminergic, cholinergic, serotonergic, adrenergic, or γ-aminobutyric acid, and D1-type receptors). Its absorption from the gastrointestinal tract is slow and even reduced by concomitant food intake, with reported peak serum levels ranging from 1 to 6 h after oral intake and average elimination half-life ranging from 3 to 10 h (Mauri et al., 1996). In higher dosages (e.g., 800–1000 mg/day), sulpiride causes antipsychotic effects, probably by equally blocking both pre- and postsynaptic receptors. In lower dosages (e.g., 50–150 mg/day), however, sulpiride exhibits a mild stimulant effect that is used for treating symptoms of depression (Mauri et al., 1996; Uchida et al., 2005). This paradoxical effect was hypothesized to be due to prevalent blockage of presynaptic autoreceptors leading to enhanced dopamine neurotransmission (Kuroki, Meltzer, & Ichikawa, 1999). In previous studies with healthy participants, single doses of sulpiride have been well tolerated, and participants were usually not able to guess whether they received sulpiride or placebo (Chavanon, Wacker, & Stemmler, 2013; Wacker, 2018; Wacker et al. 2013).

#### 3.2.2 Divergent thinking assessment

Participants completed four paper-and-pencil tasks obtained from the inventiveness scale from the Berlin Intelligence Structure Test (BIS-4; Jäger, Süß, & Beauducel, 1997): The verbal subtests *possible uses* (list as many alternate uses for a cushion as possible; AM) and *specific traits* (enumerate distinct characteristics and skills a good salesman should not have; EF) as well as the two figural-spatial subtests *symbol completion* (draw various real-life objects by completing a single figural element; ZF) and *object design* (compose real-life objects out of given figural elements; OJ). Each task was time-limited, and all instructions were read out aloud by one experimenter to assure standardized instruction times. In line with the manual’s instructions, the tasks were scored for ideational fluency (number of valid solutions) and ideational flexibility (number of categorically different valid solutions) by two independent raters.

#### 3.2.3 Personality assessment

To assess participants’ trait level of openness to experience, we administered a German version of the third edition of the NEO Personality Inventory (NEO-PI-3; revised version of the NEO-PI-R by Costa & McCrae, 2010). The five domains are assessed with 48 items each, resulting in a total of 240 items (Costa & McCrae, 2010).

#### 3.2.4 Intelligence assessment

In order to control for individual differences in cognitive ability, we estimated participants’ fluid and crystallized intelligence by administering six computer-based subtests from the Intelligence Structure Battery (INSBAT; Arendasy et al., 2012). The INSBAT provides an adaptive intelligence measurement with all subtests showing conformity to the Rasch model (Frey & Moshagen, 2015). Fluid intelligence was assessed with the subtests *numeric-inductive thinking (number series)*, *figural-inductive thinking (matrices)*, and *verbal-deductive thinking (verbal reasoning)*. Crystallized intelligence was measured using the subtests *common knowledge*, *verbal fluency*, and *word meaning* (Arendasy et al., 2012). Due to the adaptive nature of the test, the overall processing time varied between participants (*M* = 56 min, *SD* = 10.6 min).

### 3.3 Procedure

In a pretesting session, participants’ eligibility for participation was verified and self-report measures were assessed. Participants were then invited to the main experimental session in groups of four. Each experimental session started at 9:30 AM in the morning and was supervised by two out of five female experimenters. When arriving at the laboratory, participants were randomly assigned to a single cabin. After ingesting the capsule, they received a light standardized breakfast and subsequently completed six subtests of the intelligence structure battery (INSBAT), on average finishing within 1.2 h (*SD* = 0.2) and thus well before sulpiride typically reaches its maximum plasma level (after *M* = 2.3 h, *SD* = 0.37, according to Caley & Weber, 1995). About 1.4 h after medication intake (*SD* = 0.21), assessments of divergent thinking ability with four paper–pencil tasks began and lasted for around 15 min. Afterwards, seven more tasks were completed to assess implicit learning, working memory, effort expenditure, information preference, and behavior in a group discussion. The results will be reported elsewhere. In the end, participants were debriefed about their experimental condition, thanked, and compensated.

### 3.4 Statistical data analysis

As recommended by the authors of the test, the divergent thinking tasks were independently scored by two trained raters (Jäger et al., 1997). For five participants, who refrained from labeling their answers in one of the figural subtasks albeit conforming to the other tasks’ instructions, we estimated their scores by imputing the mean values across the three valid scores of each participant. Mean scores were then calculated by averaging fluency and flexibility scores across the four subtasks and then centering across the whole sample. Openness to experience scores were averaged across the 48-item openness to experience scale from the NEO-PI-3. To predict divergent thinking ability from participants’ openness scores, Substance, and the Openness × Substance interaction, linear regression analyses were computed. To control for potential effects of related traits, fluid intelligence, crystallized intelligence, and extraversion were entered as covariates into the multiple regression models. All continuous predictor variables were z-transformed within Substance groups. Furthermore, post-hoc analyses were conducted to compare performance levels of participants high versus low in openness between Substance groups. For this purpose, the sample was separated into high and low open participants by median split. Statistical analyses were implemented with R, version 3.4.3 (R Core Team, 2012). The main hypotheses and analyses were preregistered at the Open Science Framework on August 9, 2017, after the collection of 70 data sets and before accessing any of the data included in the current analyses (https://osf.io/mv4xs/register/5771ca429ad5a1020de2872e). The results of the other tasks addressed in the preregistration were part of the larger project investigating the effects of dopamine on behavioral measures and will be reported elsewhere.

### 3.5 Blindness to the psychopharmacological treatment

The majority of participants (78%) indicated in a forced-choice item as part of the post-experimental questionnaire that they assumed having received placebo. The two substance groups did not differ in the percentage of participants who guessed that they had taken the drug (sulpiride group: 21.1%, placebo group: 22.7%), χ²(1) = 0.1, *p* = 0.76. Importantly, the percentage of correct guesses was independent from substance group guess (48% correct sulpiride guesses and 50% correct placebo guesses), χ²(1) = 0.07, *p* = 0.79. Furthermore, participant’s subjective confidence in whether they had taken sulpiride was not related to the correctness of their guess (χ²(3) = 0.96, *p* = 0.81). Overall, we assume that participants were not able to guess their experimental condition above chance.

## 4 Results

### 4.1 Preliminary analyses

Prior to testing the main hypotheses, we examined potential trait differences between groups that might bias the hypothesized outcomes. The placebo group did not differ from the sulpiride group in age (*t*(181.9) = −0.85, *p* = 0.34), weight (*t*(190.5) = −0.04, *p* = 0.97), fluid intelligence (*t*(186.4) = −1.50, *p* = 0.14), crystallized intelligence (*t*(187.4) = 0.46, *p* = 0.65), or openness to experience (*t*(184.5) = 0.27, *p* = 0.79). Thus, we assume that any substance effects were not confounded with relevant trait differences between groups. The inter-rater reliability of the divergent thinking scores ranged from 0.87 to 0.98 (flexibility) and 0.96 to 1.0 (fluency), indicating high to perfect agreement among raters. Treating the four tasks as items, Cronbach’s alpha internal consistency was considerably higher for fluency (*α* = 0.72) than for flexibility (*α* = 0.61). Furthermore, fluency and flexibility scores were highly correlated, *r* = 0.86. Because flexibility contained highly redundant and less reliable information, we decided to focus the analyses on fluency scores. However, in line with the preregistered analysis plan all main analyses are also reported for flexibility. The openness to experiences scale yielded an internal consistency of *α* = 0.87. Mean openness scores in our sample (*M* = 122.6, *SD* = 17.6) were very similar to the mean of the norm sample reported in the NEO-PI-R manual for males of a similar age (*M* = 122.7, *SD* = 19.3). The aggregated openness scores were normally distributed and did not contain any outliers (i.e., more than three standard deviations from the mean). In divergent thinking, however, three participants reached fluency and flexibility scores more than three standard deviations above the mean. Since we did not specify the removal of outliers prior to analyzing the data (see preregistration link in the methods section), the data were analyzed as they are. However, additional analyses were performed without the outliers to ensure that the effects were not driven by extreme values.

### 4.2 Divergent thinking performance

**Figure 1.**
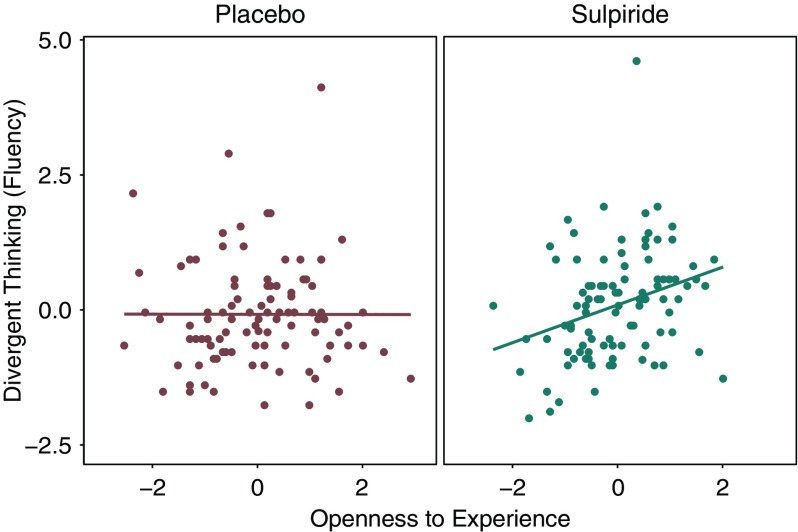
Relationship between openness to experience and divergent thinking in each substance group. Fluency scores (i.e., number of valid solutions) were z-standardized across the whole sample. Openness to experience scores were z-standardized within each experimental group.

**Figure 2.**
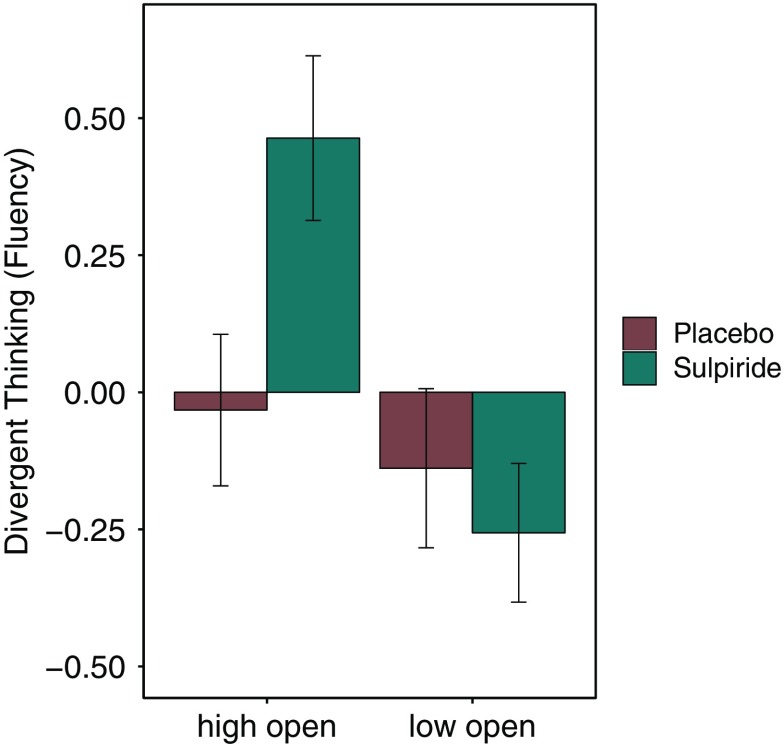
Mean divergent thinking scores separated by openness to experience and substance groups. Participants were assigned to high and low open groups by median split. Divergent thinking scores were z-standardized across the whole sample. Error bars depict standard errors of the mean (*SEM*).

Predicting fluency with openness to experience and substance group, a significant Openness × Substance interaction emerged (*b* = 0.31, *t*(189) = 2.19, *p* = 0.029). Follow-up analyses revealed a positive correlation between fluency and openness to experience within the sulpiride group (*r*(93) = 0.304, *p* = 0.0027), and a near zero correlation within the placebo group (*r*(96) = −0.002, *p* = 0.98; see Figure 1). The data did not reveal any main effects of either openness (*b* = −0.001, *t*(189) = −0.016, *p* = 0.99) or substance group (*b* = 0.166, *t*(189) = 1.18, *p* = 0.24). When conducting the analyses without outliers, the interaction remained significant (*b* = 0.34, *t*(187) *=* 2.66, *p* = 0.0086). Furthermore, the Openness × Substance interaction was also significant for flexibility, *b* = 0.29, *t*(189) = 2.05, *p* = 0.041 (without outliers: *b* = 0.32, *t*(186) = 2.47, *p* = 0.014). To examine the effects of sulpiride on mean performance levels, we compared the scores of participants high versus low in openness between substance groups. As illustrated in Figure 2, the highest scores were reached by highly open participants within the sulpiride group. Specifically, highly open participants reached significantly higher fluency scores within the sulpiride group than within the placebo group, *t*(92.8) = 2.43, *p* = 0.017 (without outliers: *t*(91.6) = 2.92, *p* = 0.0044). Less open participants did not show any significant differences as a function of substance group, *t*(91) = 0.61, *p* = 0.54 (without outliers: *t*(91) = 0.61, *p* = 0.54).

To examine the specificity of the effects to openness to experience, we additionally tested a regression model with Openness, Substance, Fluid Intelligence, Crystallized Intelligence, Openness × Substance, Fluid Intelligence × Substance, and Crystallized Intelligence × Substance. Neither fluid nor crystallized intelligence significantly interacted with substance group (*b ≤* 0.21, *t*(185) ≤ 1.44, *p ≥* 0.15) and the Openness × Substance interaction remained significant, *b* = 0.31, *t*(185) = 2.13, *p =* 0.035 (without outliers: *b* = 0.34, *t*(183) = 2.64, *p* = 0.009), indicating that the hypothesized effects were not driven by intelligence. In the total sample, openness to experience was unrelated to fluid intelligence (*r* = 0.055, *t*(191) = 0.76, *p* = 0.45) and positively correlated with crystallized intelligence (*r*(191) = 0.14, *p* = 0.045). Since openness was also positively associated with extraversion (*r*(191) = 0.25, *p* = 0.0005), we tested possible effects of extraversion in a separate model including Openness, Extraversion, Substance, Openness × Substance, and Extraversion × Substance. When extraversion was included as a predictor, the Openness × Substance interaction just failed to reach statistical significance in the analysis including the outliers, *b* = 0.28, *t*(187) = 1.9, *p* = 0.059 (without outliers: *b* = 0.31, *t*(185) = 2.36, *p* = 0.019). However, the effect observed for openness was not driven by extraversion as indicated by the nonsignificant Extraversion × Substance interaction (*b* = 0.16, *t*(189) = 1.13, *p* = 0.26).

## 5. Discussion

In the present study, we examined the effects of a pharmacological manipulation of dopamine on divergent thinking and its association with openness to experience. The dopamine receptor blocker sulpiride was administered in a placebo-controlled between-subjects design in a sample of healthy males. As expected, the dopamine manipulation moderated the relationship between openness to experience and divergent thinking. Specifically, we observed a positive correlation in the sulpiride group and a near zero correlation in the placebo group. Furthermore, highly open participants reached higher scores under sulpiride versus placebo, whereas less open individuals did not show significant differences between substance groups.

These observations are broadly consistent with the hypothesis that trait variation in openness to experience partly stems from individual differences in dopamine activity (DeYoung, 2013; DeYoung et al., 2005): Matching the empirically underpinned involvement of dopamine in creative potential (e.g., de Manzano et al., 2010; Lhommée et al., 2014) and the theorized involvement of dopamine in openness to experience (DeYoung, 2013), we expected and found that the effect of the dopaminergic agent sulpiride on divergent thinking interacts with individual differences in trait levels of openness to experience.

**Figure 3.**
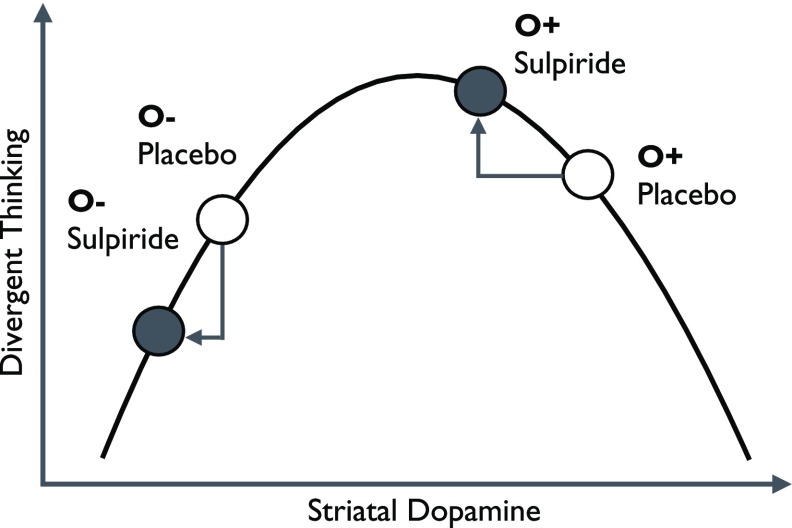
Interpretation of the current results based on the model suggested by Boot et al. (2017) linking striatal dopamine and divergent thinking via an inverted U-shaped function. O− = low trait levels of openness to experience; O+ = high trait levels of openness to experience.

According to a framework integrating creative cognition with dopaminergic modulation of fronto-striatal dopamine networks (Akbari Chermahini & Hommel, 2012; Boot, Baas, van Gaal, & De Dreu, 2017), the manipulation of striatal dopamine neurotransmission via dopaminergic substances might lead to opposing effects in healthy individuals with low versus high baseline levels of dopamine due to an inverted U-shaped relationship between striatal dopamine levels and divergent thinking. Supposing that certain personality traits are linked to differences in baseline dopamine activity, this idea implies a dependence of pharmacological dopamine effects on the personality traits in question. Supporting this claim, our results suggest that sulpiride enhanced divergent thinking only in highly open individuals, while performance levels of less open individuals were not significantly affected by the sulpiride administration. Assuming that sulpiride (200 mg) had mostly antagonistic effects in the current study, it would be conceivable that pharmacological reductions in dopamine activity caused only highly open individuals to reach an optimal striatal dopamine level, whereas less open individuals were pushed down the ascending limb of Boot et al.’s (2017) inverted U (see Figure 3). Using the indirect dopamine agonist methylphenidate in a similar design, Gvirts et al. (2017) found verbal divergent thinking (numerically) diminished in participants scoring highly on novelty seeking (i.e., a trait positively correlated to openness; Goclowska et al., 2018), but (numerically) increased in participants scoring low in novelty seeking resulting in a drug-induced cancellation of the positive association between novelty seeking and divergent thinking present under placebo. Assuming that novelty seeking, like openness, is associated with elevated levels of dopamine, methylphenidate may have pushed individuals high in novelty seeking just beyond the optimal dopamine level, whereas it moved individuals low in novelty seeking to a point just before the optimal dopamine level in the study by Gvirts et al. (2017).

While the model proposed by Boot et al. (2017) may thus potentially explain both the current findings and the earlier results by Gvirts et al. (2017), it should be noted that proposed interpretation relies on the assumption that sulpiride (200 mg) primarily acted as an *antagonist* in the present study, whereas some of Wacker’s earlier pharmacological work on extraversion was more compatible with a predominantly presynaptic effects on autoreceptors and, thus, an *agonistic* postsynaptic effect (e.g., Wacker et al., 2006, 2013). In addition, the interpretation proposed in Figure 3 leaves open the puzzling question, why the current study did not replicate the well-established association between openness to experience and divergent thinking under placebo conditions (Puryear et al., 2017). Possibly contextual factors of the present investigation like the group setting, the intelligence tests preceding the divergent thinking tasks, or the presence of two opposite-sex experimenters may have led to state increases in dopamine levels that pushed the high openness beyond the optimal level of Boot et al.’s (2017) inverted U, thereby canceling out the otherwise existing openness-related differences in divergent thinking. Of course, this suggestion remains speculative until directly tested by future work.

In order to determine the specificity of the observed effects, we also tested for potential effects of intelligence and extraversion (i.e., dimensions likewise associated with brain dopamine, e.g., Grazioplene et al., 2015; Wacker & Smillie, 2015). Although openness was positively related to both crystallized intelligence and extraversion, its interaction with Substance remained virtually unchanged after statistically controlling for either extraversion or fluid and crystallized cognitive ability. The findings are in line with the previous research, suggesting that openness to experience explains a unique proportion of variance in divergent thinking even when controlling for intelligence (Benedek et al. 2014; Silvia, 2008). They are also in line with the assumption of separable dopaminergic bases for openness and extraversion.

Ideally, future research could provide a stringent test of the model depicted in Figure 3 by comparing dopamine blockage and activation induced by varying dosages of dopaminergic agents including (but not limited to) sulpiride and methylphenidate. Additional substance groups (e.g., serotonin reuptake inhibitors) should also be examined to probe the specificity of the effects of dopamine, as opposed to other neurotransmitters. Moreover, the concurrent assessment of at least somewhat more direct indicators of dopamine (i.e., eyeblink rate) could help validate the presumed effects of dopaminergic substances and dosages. Finally, future work may also address the limitation of the current study resulting from our sample restriction to male participants. Although gender has not been identified as a correlate of divergent thinking (Baer & Kaufman, 2011), it remains to be tested whether the present results replicate in other populations.

## 6. Conclusions

Taken together, the current findings provide partial support for a modulating role of individual differences in dopaminergic neurotransmission in both openness to experience and divergent thinking. Future studies should employ even larger and more diverse samples to investigate dose–response relationships using several dopaminergic and non-dopaminergic agents and measuring eyeblink rate (or other indicators of dopamine level) in addition to divergent thinking.

Due to the data protection statement included in the informed consent of this study, data cannot be made publicly available. However, data will be shared with research collaborators upon request.
